# Related Pentacyclic Triterpenes Have Immunomodulatory Activity in Chronic Experimental Visceral Leishmaniasis

**DOI:** 10.1155/2021/6671287

**Published:** 2021-02-17

**Authors:** Jéssica Adriana de Jesus, Márcia Dalastra Laurenti, Leila Antonangelo, Caroline Silvério Faria, João Henrique Ghilardi Lago, Luiz Felipe Domingues Passero

**Affiliations:** ^1^Laboratory of Pathology of Infectious Diseases (LIM50), Department of Pathology, Medical School of São Paulo University, Av. Dr. Arnaldo, 455 Cerqueira César, São Paulo, 01246-903 SP, Brazil; ^2^Laboratório de Patologia Clínica, Departamento de Patologia, Hospital das Clinicas, Faculdade de Medicina, Universidade de São Paulo, São Paulo, SP, Brazil; ^3^Laboratório de Investigação Médica (LIM03), Hospital das Clínicas, Faculdade de Medicina, Universidade de São Paulo, São Paulo, SP, Brazil; ^4^Centre of Natural and Human Sciences, Federal University of ABC (UFABC), Santo André 09210-580, Brazil; ^5^São Paulo State University (UNESP), Institute of Biosciences, São Vicente, Praça Infante Dom Henrique s/n, 11330-900 São Vicente, SP, Brazil; ^6^São Paulo State University (UNESP), Institute for Advanced Studies of Ocean, São Vicente, João Francisco Bensdorp 1178, 11350-011 São Vicente, SP, Brazil

## Abstract

Leishmaniasis is a neglected tropical disease caused by the flagellated protozoa of the genus *Leishmania* that affects millions of people around the world. Drugs employed in the treatment of leishmaniasis have limited efficacy and induce local and systemic side effects to the patients. Natural products are an interesting alternative to treat leishmaniasis, because some purified molecules are selective toward parasites and not to the host cells. Thus, the aim of the present study was to compare the *in vitro* antileishmanial activity of the triterpenes betulin (Be), lupeol (Lu), and ursolic acid (UA); analyze the physiology and morphology of affected organelles; analyze the toxicity of selected triterpenes in golden hamsters; and study the therapeutic activity of triterpenes in hamsters infected with *L.* (*L.*) *infantum* as well as the cellular immunity induced by studied molecules. The triterpenes Lu and UA were active on promastigote (IC_50_ = 4.0 ± 0.3 and 8.0 ± 0.2 *μ*M, respectively) and amastigote forms (IC_50_ = 17.5 ± 0.4 and 3.0 ± 0.2 *μ*M, respectively) of *L.* (*L.*) *infantum*, and their selectivity indexes (SI) toward amastigote forms were higher (≥13.4 and 14, respectively) than SI of miltefosine (2.7). *L.* (*L.*) *infantum* promastigotes treated with Lu and UA showed cytoplasmic degradation, and in some of these areas, cell debris were identified, resembling autophagic vacuoles, and parasite mitochondria were swelled, fragmented, and displayed membrane potential altered over time. Parasite cell membrane was not affected by studied triterpenes. Studies of toxicity in golden hamster showed that Lu did not alter blood biochemical parameters associated with liver and kidney functions; however, a slight increase of aspartate aminotransferase level in animals treated with 2.5 mg/kg of UA was detected. Lu and UA triterpenes eliminated amastigote forms in the spleen (87.5 and 95.9% of reduction, respectively) and liver of infected hamster (95.9 and 99.7% of reduction, respectively); and UA showed similar activity at eliminating amastigote forms in the spleen and liver than amphotericin B (99.2 and 99.8% of reduction). The therapeutic activity of both triterpenes was associated with the elevation of IFN-*γ* and/or iNOS expression in infected treated animals. This is the first comparative work showing the *in vitro* activity, toxicity, and therapeutic activity of Lu and UA in the chronic model of visceral leishmaniasis caused by *L.* (*L.*) *infantum*; additionally, both triterpenes activated cellular immune response in the hamster model of visceral leishmaniasis.

## 1. Introduction

Leishmaniasis is a neglected tropical disease and a public health problem worldwide, affecting vulnerable people in 98 countries, with 12 million cases detected worldwide per year; additionally, 1 billion people live in areas at risk of transmission [1]. The most severe form of the disease is visceral leishmaniasis (VL), also known as kala-azar, and in Latin America, it is caused by *L.* (*L.*) *chagasi* or *L.* (*L.*) *infantum* [2]. VL has an incidence of 1.6 cases per 100,000 inhabitants and is considered fatal if not properly treated. The main compromised organs are the spleen, liver, and bone marrow. Hepatomegaly, splenomegaly, pancytopenia, prolonged fever, and weight loss are the main clinical signs of manifested disease [3].

The therapeutic arsenal available for the treatment of leishmaniasis is scarce, and it is based on the use of the first-line drug, pentavalent antimonial [4]. Second-line drugs, such as amphotericin B, and its liposomal formulation AmBisome, paromomycin, and more recently, miltefosine, have also been used with different degrees of effectivity worldwide [4, 5]. Additionally, all these second-line drugs have limitations, such as long duration of treatment, high costs, local and systemic side effects that include pain in the local of application, nephrotoxicity, cardiotoxicity, gastrointestinal events, and teratogenic effects [6, 7].

Based on the aforementioned comments, it becomes clear the importance of identifying new leishmanicidal drugs. According to DNDi (Drugs for Neglected Diseases initiative), new prototype drugs need to be safe and affordable. In this sense, different studies have shown that natural molecules have these characteristics and can be considered prototype antileishmanial drugs [8]. Furthermore, these natural products are active and selective toward pathogens; additionally, the selectivity can be improved after synthetic modifications [9, 10].

Triterpenes are the most representative group of phytochemicals, comprising more than 20,000 recognized compounds, and are biosynthesized in plants through the cyclization of squalene [11]. Due to the structural diversity associated with their pharmacological effects, these compounds are considered interesting candidates for the development of new drugs [12, 13]. Moreover, in some Asian countries, triterpenes are used as anti-inflammatory, analgesic, hepatoprotectant, cardiotonic agents, and sedatives [14, 15].

Betulin (Be), lupeol (Lu), and ursolic acid (UA) are pentacyclic triterpenoids widely distributed in nature and exhibit important pharmacological effects such as antioxidant, antiallergic, antipruritic, antiangiogenic, and antimicrobial agents [16–18]. Additionally, these pentacyclic triterpenes have immunomodulatory activity and depending on the dose employed and route of administration Th1-associated cytokines as well as specific mediators of inflammation can be produced by innate or acquired immune cells [19]. In visceral leishmaniasis, antigen-specific immune suppression is caused by *L.* (*L.*) *infantum* [20, 21] and thus becomes essential to identify immunomodulatory-leishmanicidal prototype drugs that are able to reverse the immunosuppression caused by parasites. Considering such inherent properties of pentacyclic triterpenes on the immunity, the scarcity of drugs available to treat leishmaniasis, the severe side effects of antimonial and amphotericin B, and the emergence of resistance in *Leishmania* sp., the present study was aimed at analyzing the activity and selectivity of Be, Lu, and UA on an American strain of *L.* (*L.*) *infantum*, as well as the possible target organelles. Additionally, the toxicity, therapeutic, and immunomodulatory activities of these triterpenes were studied in the experimental model of chronic visceral leishmaniasis.

## 2. Material and Methods

### 2.1. Chemical Analysis of the Triterpenes Be, Lu, and UA

Be, Lu, and UA triterpenes were purchased from Cayman Chemicals (USA). ^1^H and ^13^C nuclear magnetic resonance (NMR) spectra were recorded, respectively, at 300 and 75 MHz in a DPX-300 spectrometer (Bruker, USA) using deuterochloroform (CDCl_3_), deuterated dimethyl sulfoxide (DMSO-d_6_), or deuterated methanol (CD_3_OD) as solvents and internal standard (Merck, Germany). Elemental analysis was obtained in an Elemental Analyzer 2400 CHN (Perkin-Elmer, USA).

### 2.2. Animals and Ethical Considerations

Golden hamsters (*Mesocricetus auratus*), 8 weeks old, were obtained from Anilab (Paulinia, São Paulo, Brazil). This study was carried out in strict accordance with the recommendations of the guide for the Care and Use of Laboratory Animals of the Brazilian National Council of Animal Experimentation (http://www.cobea.org.br). The protocol was approved by the Ethics Committee of Animal Experiments of the Institutional Committee of Animal Care and Use at the Medical School of São Paulo University (056/16). Hamsters were housed in the Animal Experimental Institute of Tropical Medicine of São Paulo (IMT-USP), according to the standards of the Committee of Animal Welfare, and allowed access to food and water ad libitum throughout the study under a 12 h light cycle. The animals were anesthetized with intraperitoneal sodium thiopental at 1 mg/200 *μ*L (Cristália, Brazil).

### 2.3. Cytotoxicity Assay

Peritoneal macrophages from golden hamsters (*Mesocricetus auratus*) (10^6^ macrophages/well) were cultured in 96-well plates in RPMI medium supplemented with 10% of fetal bovine serum (Thermo Fisher, USA), 2 mM L-glutamine (Sigma-Aldrich, USA), 10 mM Hepes (Sigma-Aldrich, USA), 1 mM sodium pyruvate, 1% *v*/*v* nonessential amino acid solution (Thermo Fisher, USA), 10 *μ*g/mL of gentamicin (Thermo Fisher, USA), and 1000 U/mL of penicillin (Thermo Fisher, USA) (R10) along with triterpenes Be, Lu, and UA or the standard drug miltefosine (2.0 to 240 *μ*M). The plates were incubated at 37°C, 5% CO_2_, for 24 h and then centrifuged at 400 × g for 10 min at 4°C and washed 3 times, followed by addition of 9.6 *μ*M of 3-(4,5-dimethylthiazol-2-yl)-2,5-diphenyltetrazolium bromide (MTT) (Sigma-Aldrich, USA). Four hours later, 50 *μ*L of 10% sodium dodecyl sulfate (SDS) was added to each well. The plates were further incubated for 18 h and read in an ELISA reader at 595 nm. Cytotoxic concentration 50% (CC_50_) was estimated using the nonlinear regression test with GraphPad Prism 5.0 software. The index of selectivity (SI) was estimated according to Passero and collaborators [22], and essentially, it is the ratio between CC_50_ and IC_50_.

### 2.4. Promastigote Assay

Promastigote forms of *L.* (*L.*) *infantum* (MHOM/BR/72/46) were incubated in a 96-well culture plate in Schneider's medium (Sigma-Aldrich, USA) supplemented with 10% heat-inactivated fetal bovine serum and 50,000 IU/mL penicillin and 50 mg/mL streptomycin (S10), at 2 × 10^6^ promastigotes/well with the triterpenes Be, Lu, and UA or standard drug miltefosine from 2.0 to 240 *μ*M (Sigma-Aldrich, USA). The negative control group was cultivated in medium and vehicle solution (PBS plus 1% DMSO). The parasites were incubated for 24 h at 25°C, then washed with 200 *μ*L of PBS three times with centrifugation at 1200 × g, 10 min at 4°C, followed by addition of 9.6 *μ*M of MTT. Four hours later, 50 *μ*L of 10% sodium dodecyl sulfate (SDS) was added to each well [23]. The plates were further incubated for 18 h and read in an ELISA reader at 595 nm. Inhibitory concentration 50% (IC_50_) was estimated using GraphPad Prism 5.0 software.

### 2.5. Activity of Triterpenes in Intracellular Amastigotes, Nitric Oxide, and Hydrogen Peroxide Production

Peritoneal macrophages from golden hamsters (10^6^ macrophages) were collected and cultured on round coverslips in 24-well plates during 4 h in R10, and then, cells were infected with *L.* (*L.*) *infantum* promastigotes (10 parasites per 1 peritoneal macrophage). The plates were incubated overnight at 5% CO_2_ at 37°C. Then, the triterpenes Be (14 to 113 *μ*M), Lu (12 to 96 *μ*M), and UA (2 to 22 *μ*M) were added to the infected macrophages. Of note, the highest concentration used was always below the values of the respective CC_50_ values. Miltefosine (18 to 74 *μ*M) was used as the positive control. After 24 hours, macrophage supernatants were collected and stored at -80°C for quantifications of nitric oxide (NO) (Life Technologies, USA) and hydrogen peroxide (H_2_O_2_) (Life Technologies, USA) according to the manufacturer's instructions of the respective kits. The coverslips were dried at room temperature, fixed in methanol, and stained by Giemsa (Sigma-Aldrich, USA). The number of infected macrophages and of parasites per macrophage was determined at least in 100 cells. The infection index (II) was expressed as the percentage of infected macrophages multiplied by the average number of amastigotes per macrophage according to Passero and collaborators [24], and the inhibitory concentration that inhibit 50% (IC_50_) of the infection index was estimated using GraphPad Prism 5.0. As a positive control for NO and H_2_O_2_, macrophages were incubated with 100 ng/mL LPS (Sigma-Aldrich, USA) according to Passero et al. [22].

### 2.6. Ultrastructural Alterations Induced by Triterpenes in *L.* (*L.*) *infantum*

Promastigote forms of *L.* (*L.*) *infantum* (2 × 10^6^ promastigotes/well) were incubated in a 24-well culture plate in S10 with Lu and UA at EC_50_ for 24 h, 25°C. The control group was cultivated with medium and vehicle solution. The plate was centrifuged at 1200 × g, 4°C, for 10 min and washed three times with 200 *μ*L of PBS. Then, the pellets were resuspended in glutaraldehyde 2% (Sigma-Aldrich, USA), pH 7.2, and incubated at 4°C, during 60 min. Parasites were postfixed in 1% osmium tetroxide (Sigma-Aldrich, USA), and further incubated with 0.5% aqueous uranyl acetate overnight, and dehydrated in a graded series of ethanol. Then, samples were embedded in a polyester resin, thin sectioned with Reichert ultramicrotome, double stained by uranyl acetate and lead citrate (Ladd Research Industries), and examined with a Jeol 1010 (Tokyo, Japan) transmission electron microscope (TEM).

### 2.7. Cell Membrane Integrity and Mitochondrial Membrane Potential Assays

Promastigote forms of *L.* (*L.*) *infantum* (2 × 10^6^ promastigotes/well) were incubated in 96-well black culture plate (Corning Inc., USA) in S10 with the IC_50_ of triterpenes Lu and UA as well as miltefosine for 0, 10, 20, 30, 40, 50, 60, 120, and 1440 minutes. At each time point, the probes SYTOX Green at 0.5 *μ*M/well (Life Technonologies, USA) or Rhodamine 123 at 3 *μ*M/well (Sigma-Aldrich, USA) were added in the parasite culture in order to evaluate cell membrane damage or mitochondrial membrane potential, respectively. Parasites were placed in the dark for 15 minutes at 25°C, then centrifuged at 1200 g, 5 min at 10°C, and washed three times with 200 *μ*L of PBS. Plates containing parasites stained with SYTOX Green were read in a fluorescence reader using 530 nm emission wavelength and 490 nm excitation, and parasites stained with Rhodamine 123 read with 520 nm emission wavelength and 485 nm excitation. Results were normalized in relation to the control, nontreated parasites. Triton X-100 (0.05 *μ*L) (Sigma-Aldrich, USA) was used as a positive control for cell membrane damage and oligomycin at 0.1 *μ*M/well (Cayman Chemicals, USA) as positive control of mitochondrial membrane potential inhibition.

### 2.8. Hepatic and Renal Biochemical Parameters

Healthy golden hamsters (8 weeks old) were divided into four groups containing 5 animals/group. The experimental groups were arranged as follows: groups 1 and 2 were treated with 2.5 mg/kg of UA or Lu, respectively. Group 3 was treated with 5.0 mg/kg of amphotericin B [25] (Cristália, Brazil), and group 4 was constituted by animals that received only the vehicle solution (control). Animals were treated by the intraperitoneal route, once a day, during 10 days. One week after the last injection, the animals were euthanized, sera collected, and the following biochemical parameters quantified: serum alanine transaminase (ALT), aspartate aminotransferase (AST), urea, and creatinine by colorimetric method on COBAS C111 equipment (Roche, USA).

### 2.9. Therapeutic Activity of Triterpenes in Visceral Leishmaniasis

Golden hamsters were infected intraperitoneally with 2 × 10^7^*L.* (*L.*) *infantum* promastigotes (MHOM/BR/72/46). The noninfected control group was injected with PBS alone. Infected hamsters were divided into 4 groups, with 5 animals each. After 60 days of infection, animals were treated with UA, Lu, or AmB and the last group was constituted by animals that received only the vehicle solution (control). The protocol of the treatment is described in the Section 2.8. One week after the last injection, animals were euthanized and the spleen and liver were collected to quantify the splenic and hepatic parasitism by limiting-dilution assay [26]. Additionally, amastigote forms in such organs were demonstrated by immunohistochemistry technique [27].

### 2.10. Cell Immune Response

RNA from hamster spleen fragments (~10 mg) was extracted using the commercial RNeasy Mini Kit (Qiagen, Germany) according to the manufacturer's protocol. cDNA was synthesized with the SuperScript®VILO™ cDNA synthesis kit (Life Technologies, USA). Amplification consisted of an initial denaturation phase at 95°C for 10 min, followed by 40 amplification cycles consisting of 95°C for 15 s, 61°C for 90s, and 72°C for 30 s, using a thermocycler (Eppendorf, Germany). Prior to quantification, the efficiency of each reaction was verified using cDNA from the spleens of a healthy animal; it was always above 95%. Expression levels of genes of interest were normalized to *β*-actin (endogenous control). Quantitative PCR (qPCR) reaction was carried out using the GoTaq*®* 1-Step RT-qPCR System (Promega Corporation, Madison, WI, USA) and 75 nM of primers. The primer sequences (Sigma-Aldrich, USA) were as follows (5′ to 3′): IFN-*γ* forward: GACAACCAGGCCATCC and reverse: CAAAACAGCACCGACT; IL-10 forward: TGGACAACATACTACTCACTG and reverse: GATGTCAAATTCATTCATGGC; iNOS forward: CGACGGCACCATCAGAGG and reverse: AGGATCAGAGGCAGCACATC; and *β*-actin forward: TCCTGTGGCATCCACGAAACTACA and reverse: ACAGCACTGTGTTGGCATAGAGGT. Quantification results are expressed in fold changes of 2^-*Δ*Ct^ over the infected control group. PCR products were electrophoresed on 2% agarose gels to confirm amplification of products with the correct size; one single amplification product of predicted size, according to Lafuse et al. [28], was always obtained for such reactions.

### 2.11. Statistical Analysis

All data obtained have been reported as the mean of three independent assays. Values are expressed as mean ± standard error. Statistical analyses were performed using GraphPad Prism 5.0 software, and the ANOVA test was used to assess the differences between groups. Statistical significance was set at a *p* value < 0.05.

## 3. Results

### 3.1. Chemical Analysis of the Triterpenes

NMR (^1^H and ^13^C) data triterpenes betulin (Be), lupeol (Lu), and ursolic acid (UA) were recorded, and obtained data were compared with those reported in the literature [29–31]. These data, in association with elemental analysis, indicated that tested compounds exhibited more than 99.5% of purity (supplementary material 1).

### 3.2. Effect of Triterpenes on Promastigotes, Amastigotes, and Host Cells

Promastigote forms of *L.* (*L.*) *infantum* were incubated with the triterpenes during 24 h, and the inhibitory 50% concentration (IC50) for each compound was estimated. All triterpenes were active against promastigote forms of *L.* (*L.*) *infantum*, being Lu (IC50 = 4.0 ± 0.3 *μ*M) more effective at inhibiting promastigote form growth, followed by UA (IC50 = 8.0 ± 0.2 *μ*M) and Be (IC50 = 133.0 ± 3.0 *μ*M). Miltefosine, used as positive control, showed an IC50 of 13.5 ± 0.9 *μ*M (Table 1). Regarding the cytotoxic potential of triterpenes on peritoneal macrophages of golden hamsters, it was verified that Lu presented CC50 above 200 *μ*M. Triterpenes Be and UA displayed a CC50 of 170.4 ± 0.5 *μ*M and 42.1 ± 0.2 *μ*M, respectively. Miltefosine eliminated 50% of the cell population with 89.3 ± 8.3 *μ*M (Table 1).

Based on the ratio between IC_50_ and CC_50_, it was possible to calculate the SI of the triterpenes toward *L.* (*L.*) *infantum*. In this regard, compound Lu was 58.5 times more selective to promastigote forms than the host macrophages, followed by triterpenes UA and Be (SI = 5.3 and 1.3, respectively). Miltefosine presented a SI of 6.6. These data are summarized in Table 1.

Macrophages infected with *L.* (*L.*) *infantum* and treated with triterpenes Lu and UA exhibited significant decrease in the parasitism, as demonstrated by the respective IC_50_ values. As shown in Table 1, the most active triterpene was UA followed by Lu, with IC_50_ values of 3.0 ± 0.2 *μ*M and 17.5 ± 0.4 *μ*M. Be was inactive on amastigote forms of *L.* (*L.*) *infantum*. Miltefosine killed amastigote forms with an IC_50_ of 33.4 ± 2.0 *μ*M. Comparatively, Lu and UA triterpenes were more effective at killing intracellular amastigote forms compared to the standard drug miltefosine. Selectivity indexes of analyzed triterpenes over amastigote forms of *L.* (*L.*) infantum were higher than those determined to miltefosine (SI = 2.7).

### 3.3. Quantification of Nitric Oxide and Hydrogen Peroxide

**Figure 1:**
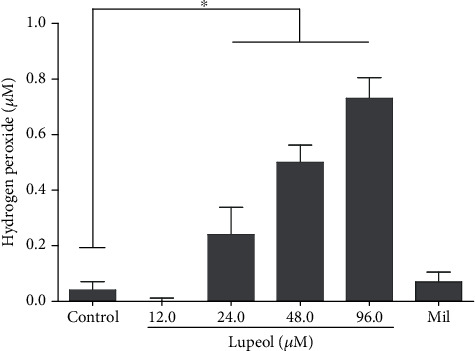
Hydrogen peroxide production. Macrophages infected with *L.* (*L.*) *infantum* were incubated with different concentrations of betulin (Be), lupeol (Lu), ursolic acid (UA), and miltefosine. After 24 h, the supernatants were collected and the NO and H_2_O_2_ levels were quantified. Only infected macrophages treated with Lu produced H_2_O_2_. ^∗^*p* < 0.05 compared to control.

Macrophages infected with *L.* (*L.*) *infantum* and treated with triterpenes Be, Lu, and UA did not produce quantifiable levels of NO. By the other side, macrophages infected and treated with Lu increased the levels of H_2_O_2_ in a dose-dependent manner when compared to the control group (*p* < 0.05), as indicated in Figure 1. Infected macrophages treated with triterpenes Be, UA, or miltefosine did not produce quantifiable H_2_O_2_. Noninfected macrophages did not produce measurable levels of H_2_O_2_. Macrophages treated with LPS produced high amounts of NO (17.1 ± 2.2 *μ*M) and H_2_O_2_ (213.1 ± 24.4 *μ*M).

### 3.4. Ultrastructural Changes in Promastigote Forms Treated with Triterpenes Lu and UA

**Figure 2:**
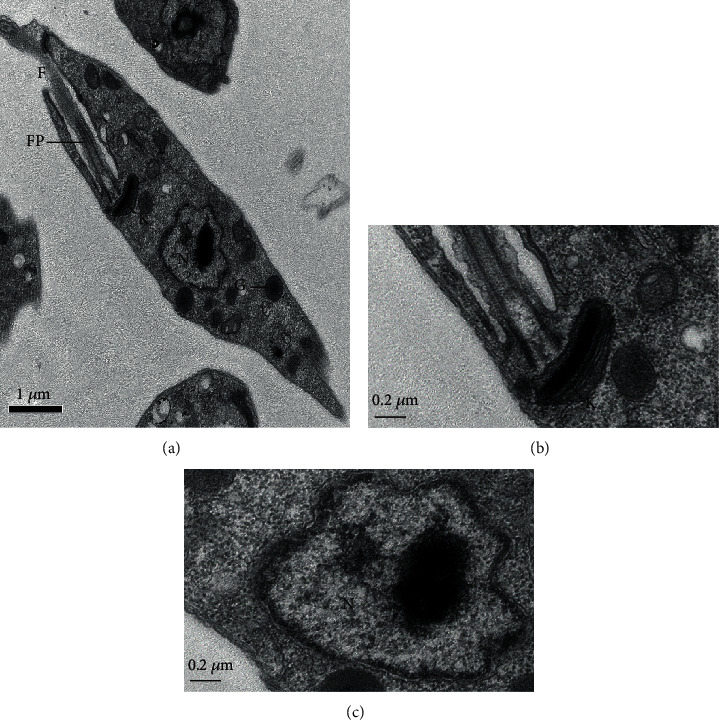
Ultrastructure of promastigote forms of *L.* (*L.*) *infantum*: (a) general morphology of *L.* (*L.*) *infantum* promastigote (×25K); (b) detail of flagellar pocket (FP) and kinetoplast (K) of control untreated (×80K); glycosome (G); (c) nucleus (N), membrane, and chromatin without changes in untreated control (×80K).

Control promastigote forms showed a well-preserved external structure, with fusiform shape and intact cell membrane (Figure 2(a)). The cytoplasm, flagellum (F), and flagellar pocket (FP) exhibited regular morphology (Figures 2(a) and 2(b)). The kinetoplast (K), presented in detail in Figure 2(b), and the nucleus (N) (Figure 2(c)) displayed normal morphology.

**Figure 3:**
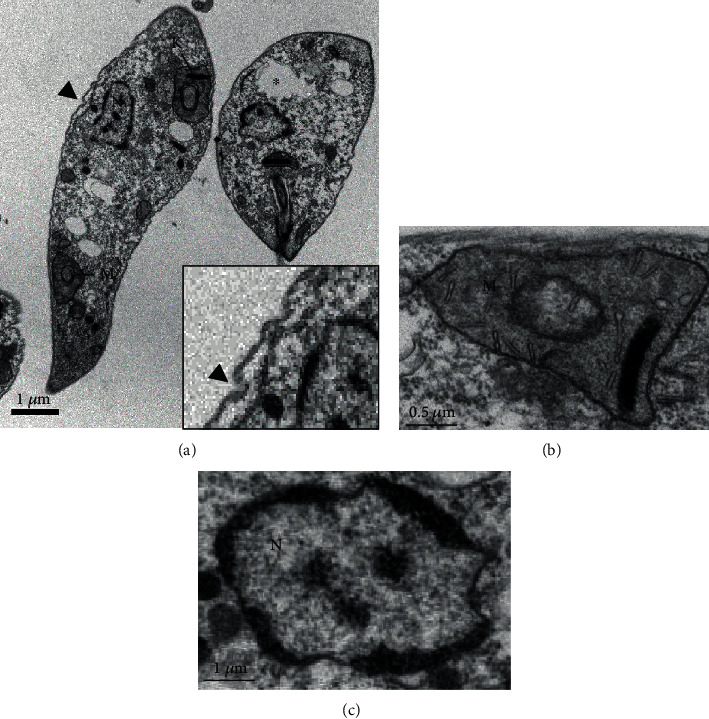
Ultrastructure of promastigote forms of *L.* (*L.*) *infantum* treated with IC_50_ of lupeol (Lu) for 24 h: (a) parasites treated with lupeol lost the fusiform shape, displayed cell membrane protrusions (arrowhead) and areas of cytoplasm degradation (^∗^); (b) detail of the mitochondria and kinetoplast from parasites treated with Lu (×50K); (c) nucleus showing condensed and fragmented chromatin after Lu treatment (×50K).

Promastigote forms treated with Lu at the IC_50_ for 24 h showed major changes in the morphology (Figure 3(a)). Cell membrane protrusions were detected (arrowhead); the cytoplasm presented areas of cytoplasm degradation (∗), resembling autophagic vacuoles (Figure 3(a)). The complex mitochondria (M)–kinetoplast (K) was swelled, and disruption of the mitochondrial cristae (Figures 3(a) and 3(b)) was observed. Parasite nucleus (N) seems to be degraded and fragmented (Figures 3(a) and 3(c)).

**Figure 4:**
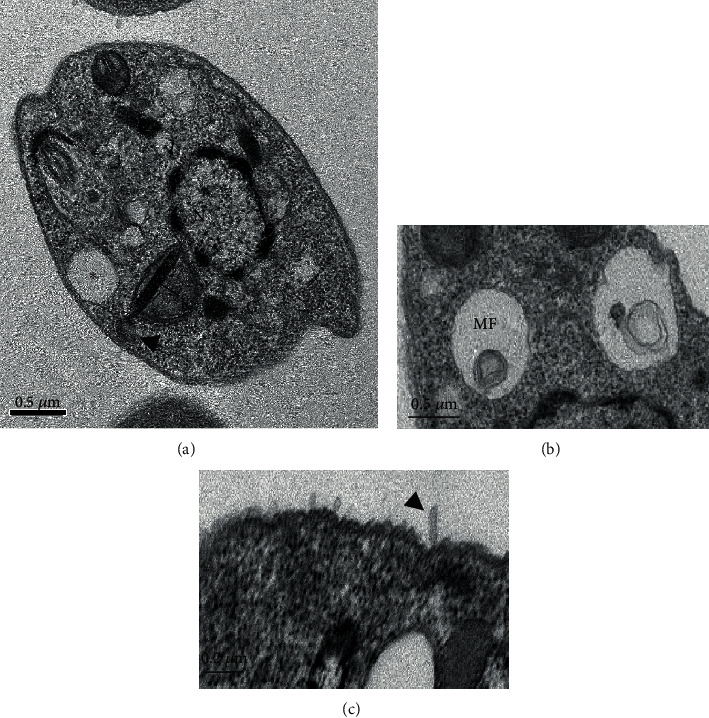
Ultrastructure of promastigote forms of *L.* (*L.*) *infantum* treated with IC_50_ of ursolic acid (UA) for 24 h: (a) parasite showing round shape morphology, with areas of cytoplasm degradation (^∗^) and nucleus with condensed and peripheral chromatin; the kinetoplast was swollen, and a bleb was identified (arrowhead) (×30K); (b) parasite treated with UA displayed myelin-like figures (MF) (×25K); (c) blebbing in the cell membrane (arrowhead) of promastigote forms treated with UA (×80K).

Figure 4(a) shows parasites treated with UA during 24 h. Parasites lost the fusiform shape (Figure 4(a)); the cytoplasm seems degraded showing changes resembling autophagic vacuoles (^∗^) (Figures 4(a) and 4(b)); myelin-like figures (MF) were detected (Figure 4(b)). Blebs were identified in the outer cell membrane of promastigote forms treated with UA (Figure 4(c), arrowhead). Moreover, mitochondrial bleb containing DNA was identified in the kDNA of parasites treated with UA (Figure 4(a), arrowhead). The nucleus (N) of promastigote forms presented with dense and peripheral chromatin that appears to be fragmented (Figure 4(a)).

**Figure 5:**
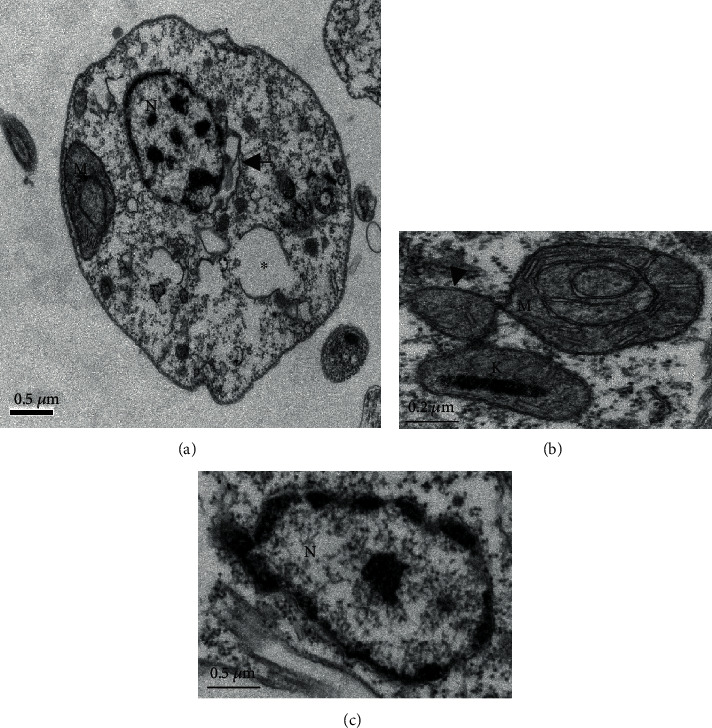
Ultrastructure of promastigote forms of *L.* (*L.*) *infantum* treated with EC_50_ of miltefosine during 24 h: (a) parasites treated with miltefosine lost its fusiform morphology; areas of cytoplasm degradation (^∗^) and detachment of nuclear membrane were observed (black arrow) (×30K); (b) miltefosine altered the morphology of the kDNA (k)-mitochondria (M) complex that presented to be fragmented and with blebs (arrowhead) (×80K); (c) nucleus (N) with condensed chromatin in parasites treated with miltefosine (×50K).

*L.* (*L.*) *infantum* treated with miltefosine lost the fusiform morphology, and the cytoplasm was degraded, showing structures resembling autophagic vacuoles (^∗^). Nuclear membrane detachment was observed (Figure 5(a), black arrow); additionally, fragmentation of chromatin was detected in the nucleus (Figures 5(a) and 5(c)). The complex kDNA-mitochondria (M) was swelled (Figure 5(a)) and fragmented, cristae were disrupted, and blebs were observed (Figure 5(b)).

### 3.5. Cell Membrane Integrity and Mitochondrial Membrane Potential Assays

**Figure 6:**
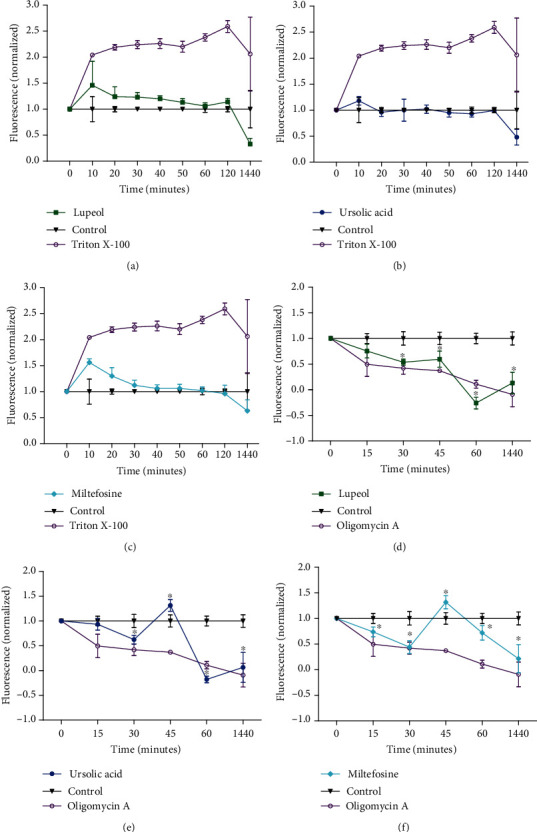
Cell membrane integrity and mitochondrial membrane potential assays of *L.* (*L.*) *infantum* promastigotes. Parasites were treated with Lu (a), UA (b), and miltefosine (c) at the IC_50_ and at different time points, the probe SYTOX® Green (0.5 *μ*M) was added, and the fluorescence intensity was analyzed. Triton X-100 was used as a positive control. In (d–f), promastigote forms were incubated with the IC_50_s of the Lu, UA, and miltefosine, respectively, and at different time points, Rhodamine 123 probe (3 *μ*M) was added to the parasites, and the fluorescence intensity was analyzed. Oligomycin A was used as a positive control. Fluorescence was normalized relative to the control parasites.

Corroborating the morphological changes, it was observed that the triterpenes Lu and UA as well as miltefosine did not damage the cell membrane of promastigote forms of *L.* (*L.*) *infantum* (Figures 6(a)–6(c)). In addition, parasites reduced the emission of fluorescence by 50% at 1440, and it is associated with the reduction of the parasite population by 50%. Parasites incubated with Triton X-100 emitted high levels of fluorescence (Figures 6(a)–6(c)).

As observed in the TEM images, treated parasites showed morphological changes in the mitochondrial-kDNA complex, and to validate such alterations, the mitochondrial membrane potential of treated parasites was analyzed. Triterpenes Lu and UA as well as positive control miltefosine (Figures 6(d)–6(f)) decreased the mitochondrial membrane potential (*ΔΨ*m) in a time-dependent manner at 30 minutes of incubation. Additionally, at 45 minutes, a hyperpolarization was detected in parasites treated with UA and miltefosine. Parasites treated with triterpenes Lu and UA displayed complete inhibition of the mitochondrial membrane potential at 60 and 1440 minutes of incubation (Figures 6(d) and 6(e), respectively). The mitochondria of parasites treated with miltefosine showed complete inhibition of the mitochondrial membrane potential at 1440 minutes (Figure 6(e)). Oligomycin (positive control) inhibited the mitochondrial membrane potential over time.

### 3.6. Hepatic and Renal Biochemical Parameters

**Figure 7:**
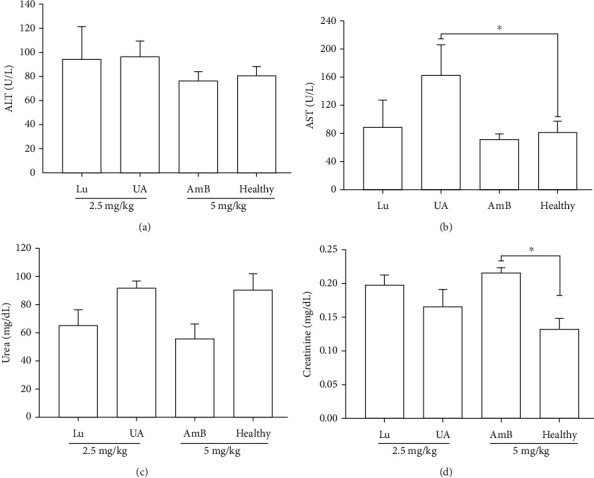
Blood biochemical parameters of hamster treated with lupeol (Lu), ursolic acid (UA), and amphotericin B (AmB). Levels of ALT (a), AST (b), urea (c), and creatinine (d) were quantified in the serum of animals treated with 2.5 mg/kg of lupeol (Lu), ursolic acid (UA), or 5 mg/kg of amphotericin B (AmB) during 10 consecutive days by intraperitoneal route. ^∗^*p* < 0.05 indicates statistical significance.

Deficiencies in the metabolism and excretion of triterpenes were analyzed by hepatic and renal functions, respectively. In respect to hepatic function, it was verified that UA treatment caused a significant increase in the level of AST (Figure 7(a)) but not ALT (Figure 7(b)). Lu and AmB did not change the biochemical parameters of the liver. Animals treated with Lu and UA did not change renal functions, as the levels of urea and creatinine were similar to the healthy group (Figures 7(c) and 7(d)). By the other side, animals treated with AmB significantly increased the level of serum creatinine, as demonstrated in Figure 7(d).

### 3.7. Therapeutic Activity of Triterpenes in Visceral Leishmaniasis

**Figure 8:**
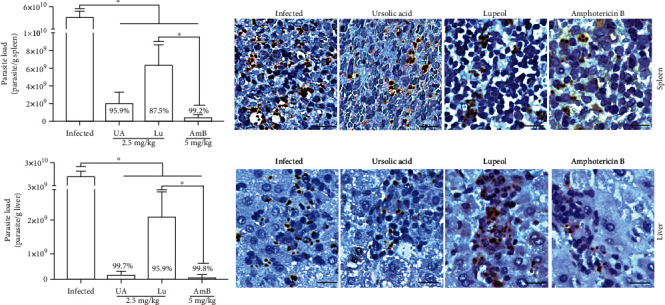
Splenic and hepatic parasitism from hamsters infected with *L.* (*L.*) *infantum* and treated with triterpenes. Hamsters were infected with promastigote forms of *L.* (*L.*) *infantum*, and four weeks after infection, animals were intraperitoneally treated once daily during 10 days with 2.5 mg/kg of ursolic acid (UA), lupeol (Lu), or 5 mg/kg of amphotericin B (AmB). The splenic and hepatic parasitism was quantified by limiting-dilution assay; additionally, amastigote forms were stained by immunohistochemistry technique.

Infected hamsters treated with 2.5 mg/kg of UA or Lu showed significant reduction in the splenic and hepatic parasitism (Figures 8(a) and 8(b), respectively) in comparison to infected control (*p* < 0.05), as demonstrated by limiting-dilution assay. In addition, tissue amastigote forms were stained by immunohistochemistry, and it is possible to observe that the treatment carried out with UA or Lu drastically decreased the number of parasites in comparison to the spleen and liver of infected control.

### 3.8. Cell Immune Response

**Figure 9:**
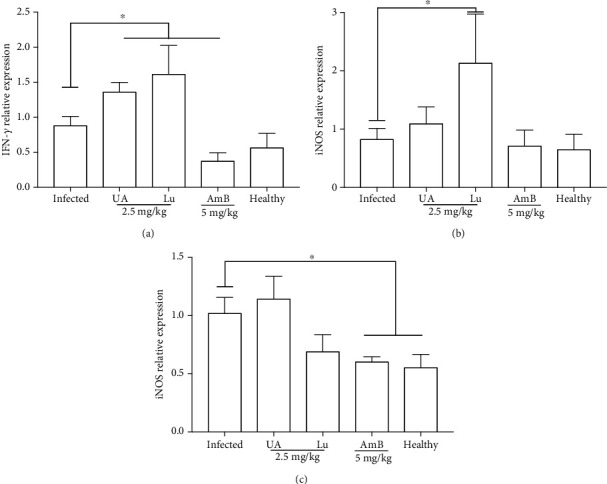
Cell immune response of hamsters infected with *L.* (*L.*) *infantum* and treated with triterpenes and amphotericin B. IFN-*γ* (a), iNOS (b, and IL-10 (c) relative gene expression in the spleen of infected golden hamsters treated with 2.5 mg/kg of ursolic acid (UA) or lupeol (Lu) and 5 mg/kg of amphotericin B (AmB). Relative gene expression was estimated by quantitative PCR. The expression levels of genes of interest were normalized to *β*-actin. ^∗^*p* < 0.05 indicates statistical significance.

Analysis of cellular immune response in the spleen of *L.* (*L.*) *infantum*-infected hamsters showed that animals treated with 2.5 mg/kg of UA or Lu expressed higher levels of IFN-*γ* compared to the infected control (Figure 9(a), *p* < 0.05). Animals treated with amphotericin B expressed lower levels of IFN-*γ* in comparison to the infected control, and it was similar to the healthy control. In respect to iNOS gene expression, only animals treated with lupeol showed significant elevation in the expression of this mediator of inflammation compared to the infected control (Figure 9(b), *p* < 0.05). Animals treated with 5 mg/kg of amphotericin B exhibited a significant decrease expression of IL-10 in comparison to the infected control (Figure 9(c), *p* < 0.05).

## 4. Discussion

The quality of drugs is a fundamental factor to ensure pharmacological activity and minimize the occurrence of unwanted effects resulting from the presence of impurities and/or degradation products. Thus, according to the nuclear magnetic resonance, associated with the elemental analysis, it was possible to confirm the purity degree of all studied triterpene (see supplementary material 1). Additionally, NMR spectral analysis was useful to confirm the identity of triterpenes such as betulin (Be), lupeol (Lu), and ursolic acid (UA). These triterpenes showed *in vitro* leishmanicidal activity on promastigote forms of *L.* (*L.*) *infantum* that were more sensitive to Lu, followed by UA and Be. However, Lu and UA impacted the survival of intracellular amastigote forms, while Be was inactive. Although triterpenes presented similar structures, they also displayed different activities on promastigote and amastigote forms. Possibly, the liposolubility [32] is one factor associated with the biological activities found herein, since to interact with the cell membrane, molecules need to present hydrophobic groups, allowing their uptake by the cell [33]. The lipophilicity of a compound can be characterized by its partition coefficient (log*P*) between octanol and water, octanol being assumed to have a similar lipophilicity to cell membranes [32]. This coefficient may be used as one of the predictors of drug absorption by passive diffusion [34]. Lu is the triterpene with the highest lipophilicity, with a log *P* of 7.67 [35], followed by the UA and Be with log *P* values determined, respectively, as 6.43 [36] and 6.17 [32], suggesting that these triterpenes have intermediate lipophilicity to interact with the cell membranes and access to the intracellular environment.

Additionally, minor structural differences among studies triterpenes can determine the potency of leishmanicidal effect. Considering Be structure, the presence of two hydroxyl groups at positions C-3 and C-28 should be crucial to the absence of the leishmanicidal activity of this triterpene. By the other side, Lu exhibits only one hydroxyl group at position C-3 and it is 33 times more effective at killing promastigote forms than Be. Additionally, on amastigote forms, this difference is higher, since Be was inactive, while Lu showed high activity and selectivity toward intracellular forms. UA was the most active at killing amastigote forms (5.8 times) in comparison to Lu, and such pharmacological activity has been related to the presence of the carboxylic acid at the position C-28 in the structure of compound UA that was not observed in the structure of Lu. By comparing the structure of all studied triterpenes, the presence of the hydroxyl group at the position C-28 found in the structure of Be may determine the low or absent leishmanicidal activity found herein. Additionally, it is important to note that Lu and UA showed higher activity and selectivity toward parasites than miltefosine, and thus, these molecules should be considered to develop new leishmanicidal drugs. Additionally, previous studies have been verified that both Lu and UA are active on promastigote and amastigote forms of different *Leishmania* species [26, 37–39], suggesting that besides high selectivity, both triterpenes have multispectral activity that needs to be considered for drug development.

Regarding cytotoxic potential of triterpenes on peritoneal macrophages of golden hamsters, it is important to note that Lu had CC_50_ above 240 *μ*M that was higher than that of miltefosine. UA showed the highest toxic potential toward macrophage, and it was associated with the presence of carboxylic acid at C-28 and hydroxyl group at C-3 position. In spite of showing some toxicity to the host cell, the selectivity toward amastigote forms (SI~14) suggests that this triterpene is still an important leishmanicidal agent. Moreover, other studies have already shown that these triterpenes have absent or low cytotoxic effects on different cell lineages [40–43], suggesting the applicability of such molecules in therapeutic studies.

During infection, *Leishmania* sp. suppresses respiratory burst in the host macrophages, so compounds able to increase macrophage respiratory activity can aid the parasite elimination. In this regard, it was observed that infected macrophages treated with Lu produced elevated amounts of H_2_O_2_ that is able to trigger programmed cell death in *Leishmania* sp. [42, 44, 45]. Thus, Lu was able to activate infected host macrophages to a leishmanicidal state, and such activity, triggered by Lu, can be an additive mechanism to eliminate parasites [41].

In order to analyze major morphological changes induced by triterpenes, promastigote forms of *L.* (*L.*) *infantum* were analyzed by transmission electron microscopy, followed by physiological changes in the plasma and mitochondrial membranes. In general, parasites showed altered morphology, intracellular disorganization, blebs, chromatin condensation, nuclear fragmentation, and different cytoplasmic alterations, mainly related to the cytoplasmic extraction. Additionally, changes in the parasite mitochondria and nucleus suggest that triterpenes act on the parasite's bioenergetics or even inducing programmed cell death [46–48]. In fact, Lu and UA were able to inhibit mitochondrial transmembrane potential after 30 minutes of incubation, suggesting that the mitochondria can be the target organelle of these triterpenes. Additionally, parasites treated with UA showed a transient recovery of the mitochondrial membrane potential that may be associated with a hyperpolarization of this organelle as previously mentioned by Bilbao-Ramos and collaborators [38], suggesting that UA triggers programmed cell death in *L.* (*L.*) *infantum*. According to Vannier-Santos and Castro [49], pyknosis and nuclear fragmentation observed in parasites treated with drugs can be considered a morphological indicator of programmed cell death. In parasites incubated with miltefosine, shrinkage of the cell surface was observed, presence of myelin-like figures, and fragmentation of mitochondria and nuclear DNA suggests the occurrence of programmed cell death, as previously demonstrated [50, 51], suggesting that miltefosine can be a good control of programmed cell death in *Leishmania* sp. Previous studies have shown that programmed cell death may be a common mechanism of cell death in *Trypanosoma* sp. [52–54] and *Leishmania* [53, 55] in response to chemotherapeutic agents such as miltefosine [50] and triterpenes [39, 42, 43]. Thus, the leishmanicidal activity of triterpenes can be associated with the major changes in the mitochondria, in the nucleus, and in the intracellular compartments that might be related to programmed cell death in *L.* (*L.*) *infantum*.

Despite cell protrusions and blebs observed in the cell membrane of promastigote forms treated with Lu or UA, cell membrane lysis was not detected by morphology and physiology studies. Protrusions and blebs observed in the cell membrane of treated parasites can be the effect of the triterpenes on the cytoskeleton of promastigote forms [49]. Similar morphological changes were also observed in *Trypanosoma cruzi* treated with lysophospholipid analogues, such as edelfosine, ilmofosine, and miltefosine [56]. In addition to these changes, membrane debris were observed in areas resembling autophagic vacuoles. Previous works demonstrated that parasites recycle abnormal structures during an autophagic process [49, 57, 58]; besides targeting mitochondria, both triterpenes may also trigger autophagy in promastigote forms.

Both triterpenes showed promising *in vitro* activity and selectivity toward *L.* (*L.*) *infantum* amastigotes, and thus, golden hamsters were injected with Lu or UA to evaluate hepatic and renal functions after treatment. Healthy golden hamsters treated with UA showed a significant increase of aspartate transaminase (AST) compared to the healthy animals. Both ALT and AST are produced by hepatocytes; therefore, increased levels of ALT and AST indicate hepatocellular injury. In the present study, UA-treated hamsters showed elevation only in AST, suggesting an initial hepatocellular injury. Previous studies already showed that low doses of UA are safe for BALB/c mice, and golden hamster treated with 2 mg/kg showed no changes in serum ALT or AST; furthermore, no morphological changes in the liver structure were observed; however, higher doses of UA (150 mg/kg) administered with a diet over 6 weeks can cause hepatic damage [59]. By the other side, UA can be found in fruits and vegetables present in the human diet; furthermore, this triterpene also is used as a dietary supplement for humans [60]. Thus, UA hepatotoxicity may be related to the dosage, duration of treatment, and route of administration. In contrast, Lu did not cause changes in the levels of ALT or AST.

Golden hamsters treated with Lu or UA did not change the levels of urea or creatinine, suggesting that both triterpenes are not nephrotoxic. By the other side, animals treated with 5 mg/kg of amphotericin B showed an increase in the creatinine level, suggesting that AmB caused renal toxicity in golden hamsters. Previous study already showed that hamsters treated with 5.0 mg/kg/day of AmB during 16 days displayed morphological changes compatible with acute renal toxicity [26]. AmB also induced nephrotoxicity in patients that presented reduced glomerular filtration rate and tubular dysfunction [61], reinforcing that one of the main side effects of AmB in vertebrates is renal failure.

Although the treatment with UA at 2.5 mg/kg induced toxicity to the liver, its efficacy in the model of visceral leishmaniasis was assessed, since only the levels of AST marker increased after treatment. In fact, UA and Lu were able to eliminate amastigotes forms in the spleen and liver of *L.* (*L.*) *infantum-*infected hamsters; however, UA treatment showed higher efficacy at eliminating hepatic parasites than Lu treatment; moreover, UA showed similar efficacy than AmB treatment. Lu, as well as UA, was poorly investigated concerning the leishmanicidal effect, and few available works showed the therapeutic activity of this triterpene only in the murine model of visceral leishmaniasis that mimics the acute phase of infection; thus, currently, this is the first work showing that Lu is able to decrease amastigote forms in the spleen and liver from hamsters in the chronic phase of visceral leishmaniasis.

In leishmaniasis, resistance to infection has been associated with the development of a Th1 immune response, with remarkable amounts of interferon gamma (IFN-*γ*) cytokine that can be mainly produced by NK and T cells [62–64]. As phagocytes are exposed to IFN-*γ*, classical activation and induction of microbicidal mechanisms can occur, with the participation of inducible nitric oxide synthase enzyme (iNOS), as well as nitric oxide (NO), a mediator of inflammation, that along with other reactive oxygen and nitrogen species can kill intracellular parasites [64–66]. In the present study, an association between leishmanicidal activity *in vivo* and increased IFN-*γ* expression was observed, suggesting that both these triterpenes may direct the immune response to a Th1 immune response, emphasizing that besides a direct activity on parasites, the therapeutic activity of triterpenes is mediated by immune response activation [26, 39].

In contrast to resistance, the susceptibility in visceral leishmaniasis has been accompanied by elevation in the levels of IL-10, a cytokine with anti-inflammatory activity and suppressive effects on the Th1 immune response. Thus, elevation in the level of IL-10 frequently results in parasite proliferation as well as chronification of *Leishmania* infection [67, 68]. UA or Lu treatment did not inhibit IL-10 expression. Possibly, in this chronic model of visceral leishmaniasis, where the disease is fully manifested, both Lu and UA lose the ability to restrain IL-10 expression and/or production, as mentioned previously [26], maintaining a small number of parasites in the spleen and liver of hamsters. Despite that, it is still important to note that both triterpenes eliminated high amounts of amastigote forms in the spleen and liver of infected animals, and even Lu is being less potent than UA and AmB, it was not toxic for hamsters and should be viewed as an important target to develop new leishmanicidal drugs.

Previous studies already discussed the *in vitro* and *in vivo* activities of both Lu and UA [26, 37, 69]; however, this is the first comparative work showing the leishmanicidal activity of related pentacyclic triterpenes in association with their structures, as well as morphological and physiological changes that took place in *L.* (*L.*) *infantum*. In addition, this is the first work showing the comparative therapeutic activity of both Lu and UA in the experimental model of chronic visceral leishmaniasis that is the most suitable model of natural infection. Taken together, data showed herein demonstrates that UA and Lu triterpenes were able to promote effective and selective antileishmanial activity on promastigote and amastigote forms of *L.* (*L.*) *infantum*, and possibly, the parasite mitochondria may be the target organelle, since impairment of transmembrane potential was observed after 30 minutes of incubation; furthermore, morphological studies showed a complete disorganization of such organelle. Both triterpenes increased the cellular immune response in hamsters with visceral leishmaniasis, and it was associated with amastigote elimination in the spleen and liver, suggesting that the leishmanicidal activity is mediated by immunomodulation of both innate and acquired immune systems.

## Figures and Tables

**Table 1:** Leishmanicidal and cytotoxic activity and selective index of betulin (Be), lupeol (Lu), and ursolic acid (UA). Results are expressed by mean and standard error of triplicates from three different experiments.

Compound	IC_50_ (*μ*M) promastigotes	IC_50_ (*μ*M) amastigotes	CC_50_ (*μ*M)	SI promastigotes	SI amastigotes
Be	133.0 ± 3.0	NA	170.4 ± 0.5	1.3	NA
Lu	4.0 ± 0.3	17.5 ± 0.4	≥240	≥58.5	≥13.4
UA	8.0 ± 0.2	3.0 ± 0.2	42.1 ± 0.2	5.3	14.0
Miltefosine	13.5 ± 0.9	33.4 ± 2.0	89.3 ± 8.3	6.6	2.7

## Data Availability

The data used to support the findings of this study are available from the corresponding author upon request.

## Supplementary Materials

Supplementary Materials Figure 1: molecular structure of betulin, lupeol, and ursolic acid. Table 1: ^13^C NMR of botulin, lupeol, and ursolic acid. Figure 2: ^1^H NMR spectrum (*δ*, CDCl_3_+CD_3_OD, 300 MHz) of betulin (Be). Figure 3: ^13^C NMR spectrum (*δ*, CDCl_3_+CD_3_OD, 75 MHz) of betulin (Be). Figure 4: ^1^H NMR spectrum (*δ*, CDCl_3_+CD_3_OD, 300 MHz) of lupeol (Lu). Figure 5: ^13^C NMR spectrum (*δ*, CDCl_3_+CD_3_OD, 75 MHz) of lupeol (Lu). Figure 6: ^1^H NMR spectrum (*δ*, DMSO-d_6_, 300 MHz) of ursolic acid (UA). Figure 7: ^13^C NMR spectrum (*δ*, DMSO-d_6_, 75 MHz) of ursolic acid (UA). Table 2: elemental analysis results.
