# Supplementary material for: Related Pentacyclic Triterpenes Have Immunomodulatory Activity in Chronic Experimental Visceral Leishmaniasis
Source: J Immunol Res. 2021 Feb 17;2021:6671287. doi: 10.1155/2021/6671287 (PMC7906800; doi:10.1155/2021/6671287)
Supplement: Supplementary Materials — Figure 1: molecular structure of betulin, lupeol, and ursolic acid. Table 1: 13C NMR of botulin, lupeol, and ursolic acid. Figure 2: 1H NMR spectrum (δ, CDCl3+CD3OD, 300 MHz) of betulin (Be). Figure 3: 13C NMR spectrum (δ, CDCl3+CD3OD, 75 MHz) of betulin (Be). Figure 4: 1H NMR spectrum (δ, CDCl3+CD3OD, 300 MHz) of lupeol (Lu). Figure 5: 13C NMR spectrum (δ, CDCl3+CD3OD, 75 MHz) of lupeol (Lu). Figure 6: 1H NMR spectrum (δ, DMSO-d6, 300 MHz) of ursolic acid (UA). Figure 7: 13C NMR spectrum (δ, DMSO-d6, 75 MHz) of ursolic acid (UA). Table 2: elemental analysis results. [file 6671287.f1.docx]

**Figure 1 – Molecular structure of betulin, lupeol and ursolic acid**


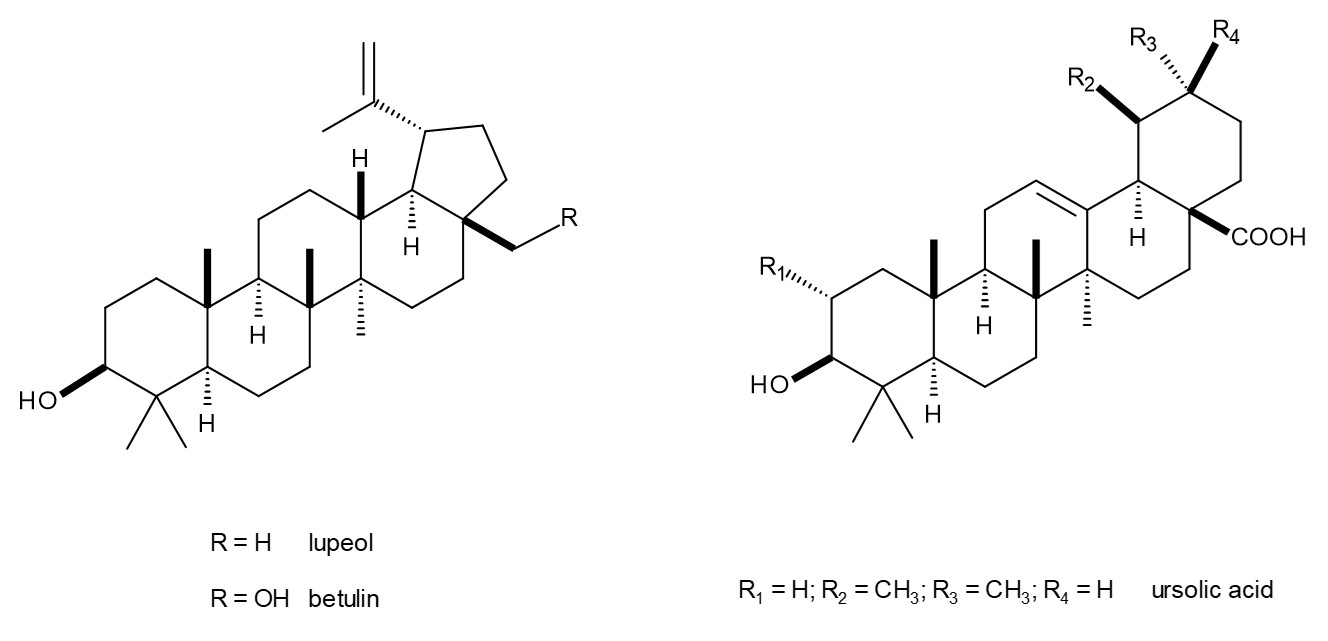


**Table 1 - ^13^C NMR of botulin, lupeol and ursolic acid**

|  | **Betulin** | |  | **Lupeol** | |  |  | **Ursolic Acid** | |
| --- | --- | --- | --- | --- | --- | --- | --- | --- | --- |
|  | δC | δC |  | δC | δC |  |  | δC | δC |
| Position | Observed ^a^ | Literature ^a^ |  | Observed ^a^ | Literature ^a^ |  |  | Observed ^b^ | Literature ^a^ |
| 1 | 38.7 | 38.8 |  | 38.6 | 38.7 |  |  | 38.7 | 38.7 |
| 2 | 27.3 | 27.2 |  | 27.4 | 27.4 |  |  | 23.5 | 23.5 |
| 3 | 78.9 | 78.9 |  | 79.0 | 78.9 |  |  | 77,0 | 79.0 |
| 4 | 38.9 | 38.9 |  | 38.9 | 38.8 |  |  | 39.6 | 39.6 |
| 5 | 55.3 | 55.3 |  | 55.3 | 55.3 |  |  | 56.3 | 56.3 |
| 6 | 18.3 | 18.3 |  | 18.3 | 18.3 |  |  | 19.0 | 19.0 |
| 7 | 34.2 | 34.3 |  | 34.3 | 34.2 |  |  | 33.4 | 33.4 |
| 8 | 40.9 | 40.9 |  | 40.8 | 40.8 |  |  | 40.0 | 40.0 |
| 9 | 50.4 | 50.4 |  | 50.4 | 50.4 |  |  | ֊ | ֊ |
| 10 | 37.2 | 37.2 |  | 37.2 | 37.1 |  |  | 38.5 | 38.5 |
| 11 | 20.8 | 20.9 |  | 20.9 | 20.9 |  |  | 23.8 | 23.8 |
| 12 | 25.2 | 25.3 |  | 25.1 | 25.1 |  |  | 125.8 | 125.8 |
| 13 | 37.3 | 37.3 |  | 38.1 | 38.0 |  |  | 138.0 | 138.0 |
| 14 | 42.7 | 42.7 |  | 42.8 | 42.8 |  |  | 42.2 | 42.2 |
| 15 | 27.0 | 27.0 |  | 27.4 | 27.4 |  |  | 29.0 | 29.0 |
| 16 | 29.2 | 29.2 |  | 35.6 | 35.5 |  |  | 24.0 | 24.0 |
| 17 | 47.8 | 47.8 |  | 43.0 | 43.0 |  |  | 47.8 | 47.8 |
| 18 | 48.8 | 48.8 |  | 48.3 | 48.0 |  |  | 52.7 | 52.7 |
| 19 | 50.4 | 47.8 |  | 47.9 | 47.9 |  |  | 38.7 | 38.7 |
| 20 | 150.5 | 150.6 |  | 150.9 | 150.9 |  |  | 38.4 | 38.4 |
| 21 | 29.8 | 29.8 |  | 29.8 | 29.8 |  |  | 27,0 | 27.0 |
| 22 | 34.2 | 34,0 |  | 40.0 | 40.0 |  |  | 37,0 | 37.0 |
| 23 | 28.0 | 28.0 |  | 28.0 | 28.0 |  |  | 27.7 | 27.7 |
| 24 | 15.4 | 15.4 |  | 15.4 | 15.4 |  |  | 15.2 | 15.2 |
| 25 | 16.1 | 16.1 |  | 16.1 | 16.1 |  |  | 15.5 | 15.5 |
| 26 | 16.0 | 16.0 |  | 15.9 | 15.9 |  |  | 17.5 | 17.5 |
| 27 | 14.8 | 14.8 |  | 14.5 | 14.5 |  |  | 24.0 | 24.0 |
| 28 | 60.5 | 60.2 |  | 18.0 | 18.0 |  |  | 181.1 | 181.6 |
| 29 | 109.7 | 109.6 |  | 109.3 | 109.3 |  |  | 16.5 | 16.5 |
| 30 | 19.1 | 19.1 |  | 19.3 | 19.3 |  |  | 21.3 | 21.3 |

^a^ CDCL_3;_ ^b^ DMSO-d_6;_

- data not identified in the experiment

**Figure 2 - ^1^H NMR spectrum (δ, CDCl_3_ + CD_3_OD, 300 MHz) of betulin (Be)**


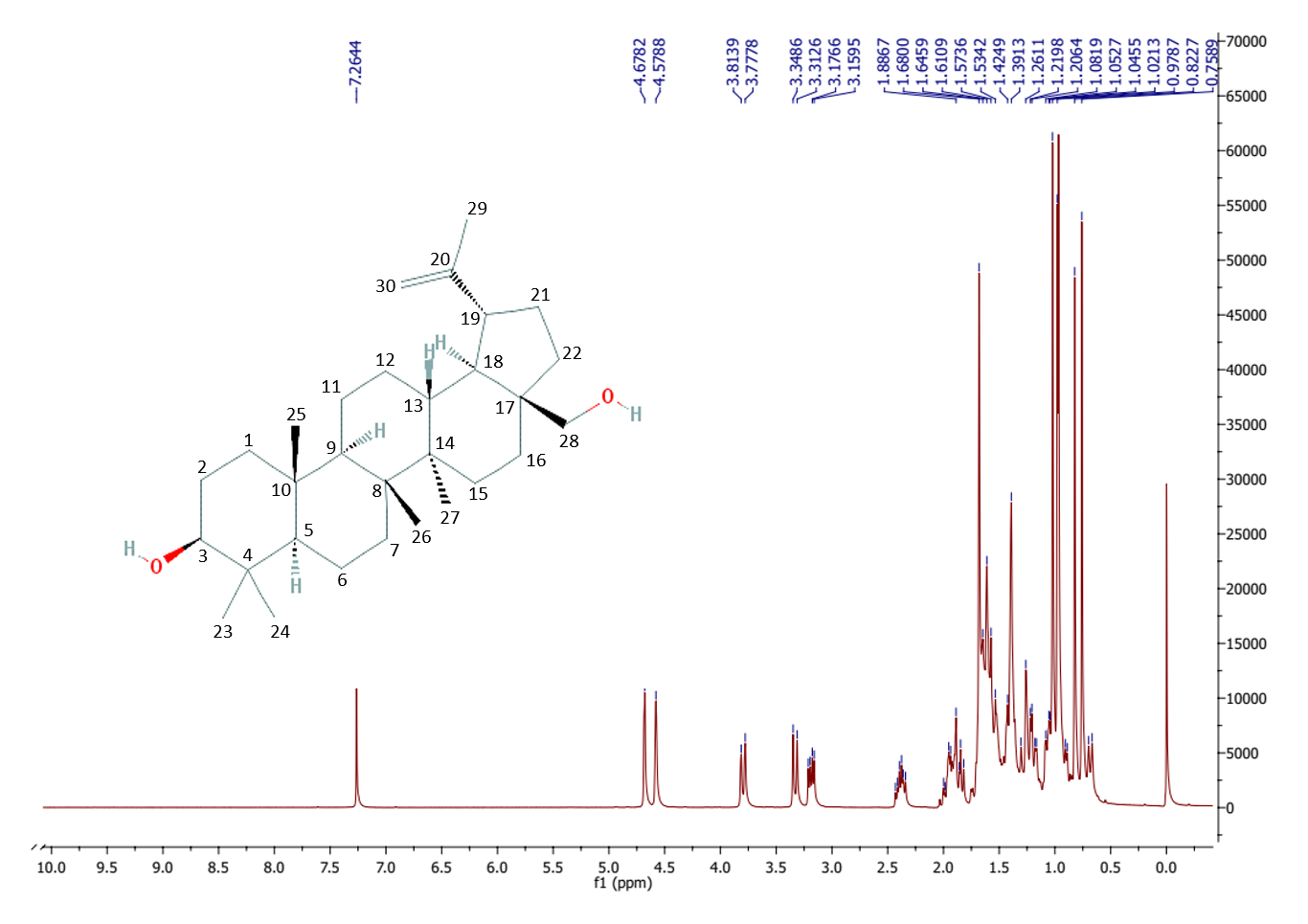


**Figure 3 - ^13^C NMR spectrum (δ, CDCl_3_ + CD_3_OD, 75 MHz) of betulin (Be)**


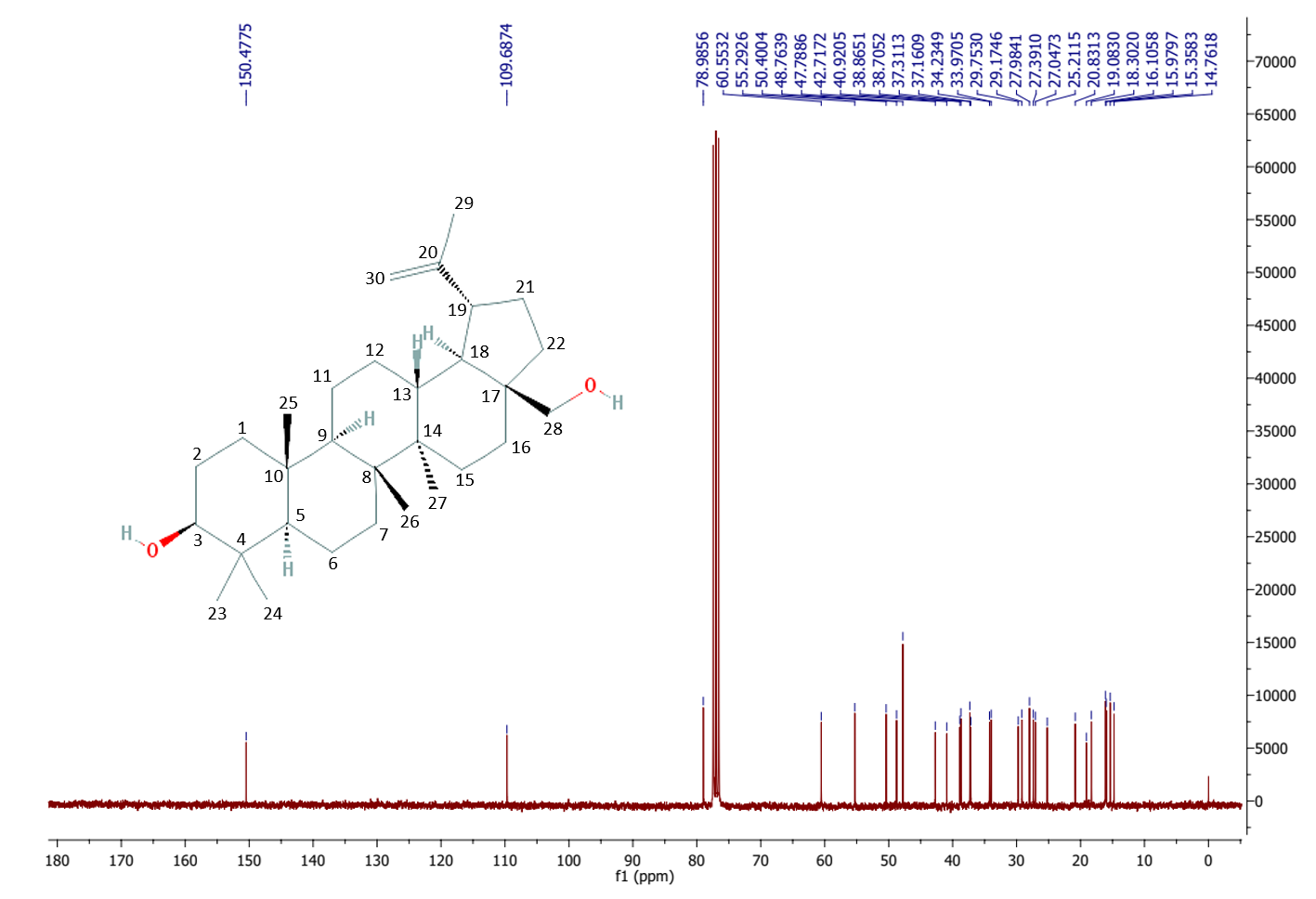


**Figure 4 - ^1^H NMR spectrum (δ, CDCl_3_ + CD_3_OD, 300 MHz) of lupeol (Lu)**


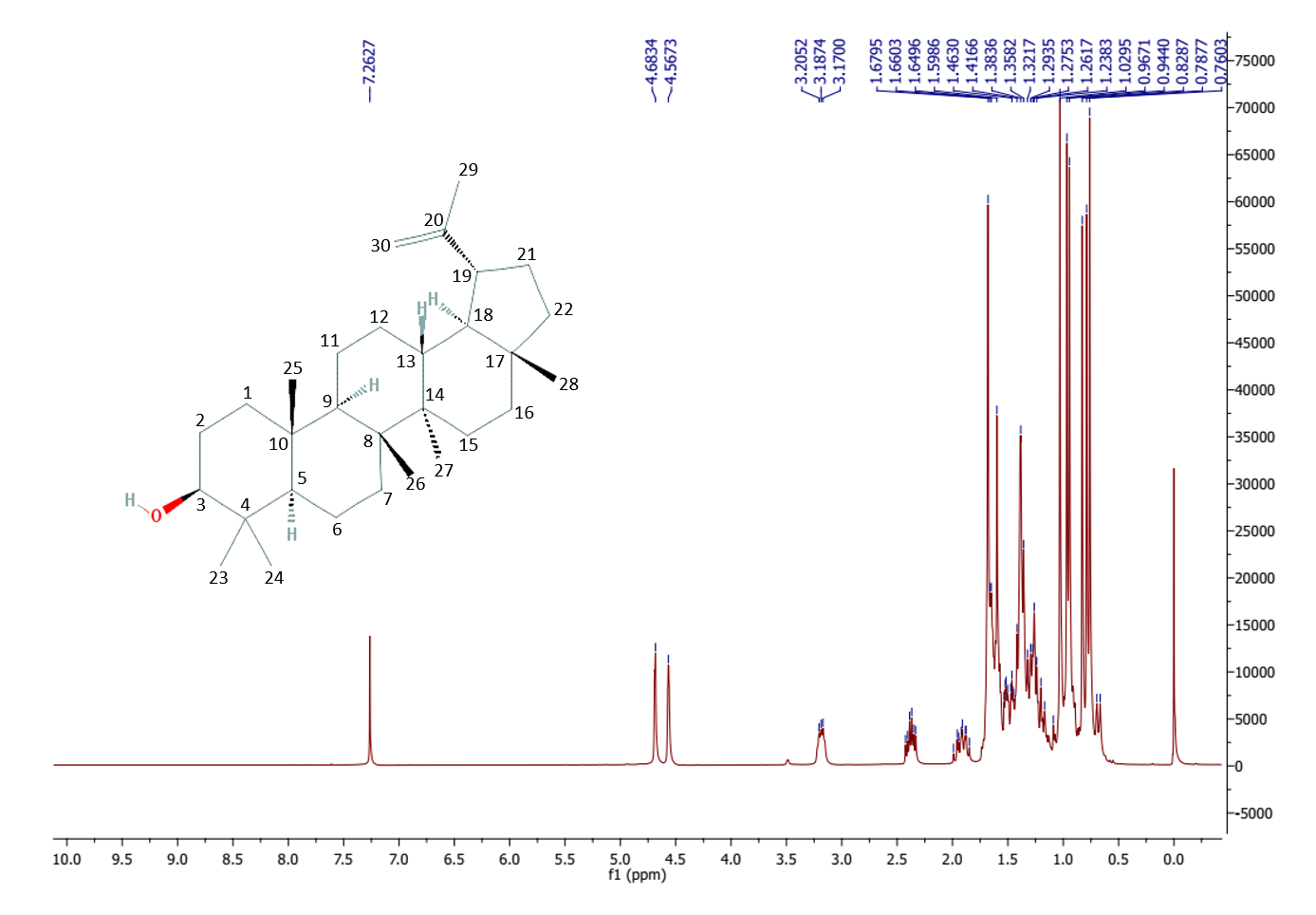


**Figure 5 - ^13^C NMR spectrum (δ, CDCl_3_ + CD_3_OD, 75 MHz) of lupeol (Lu)**


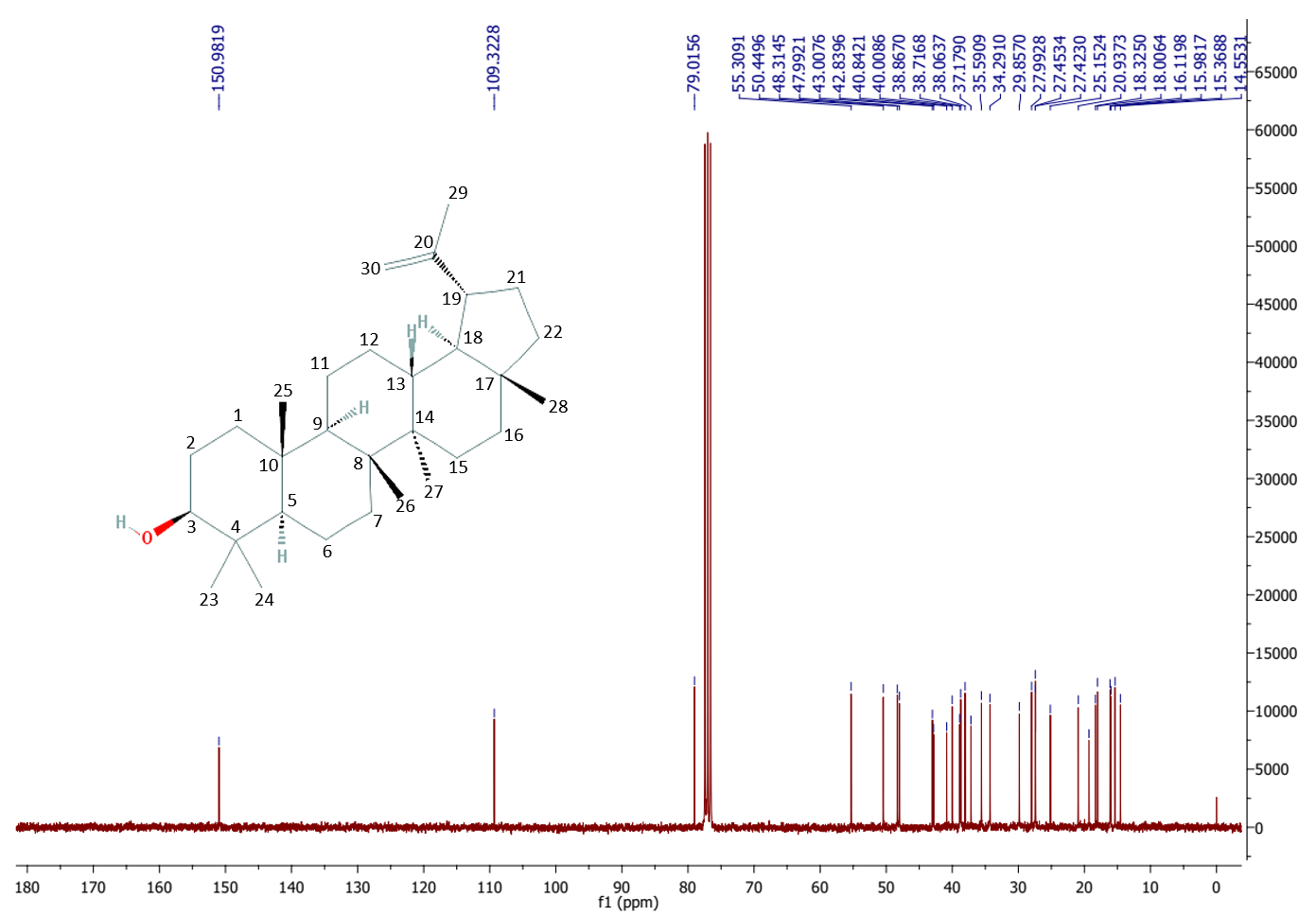


**Figure 6 - ^1^H NMR spectrum (δ, DMSO-d_6_, 300 MHz) of ursolic acid (UA)**


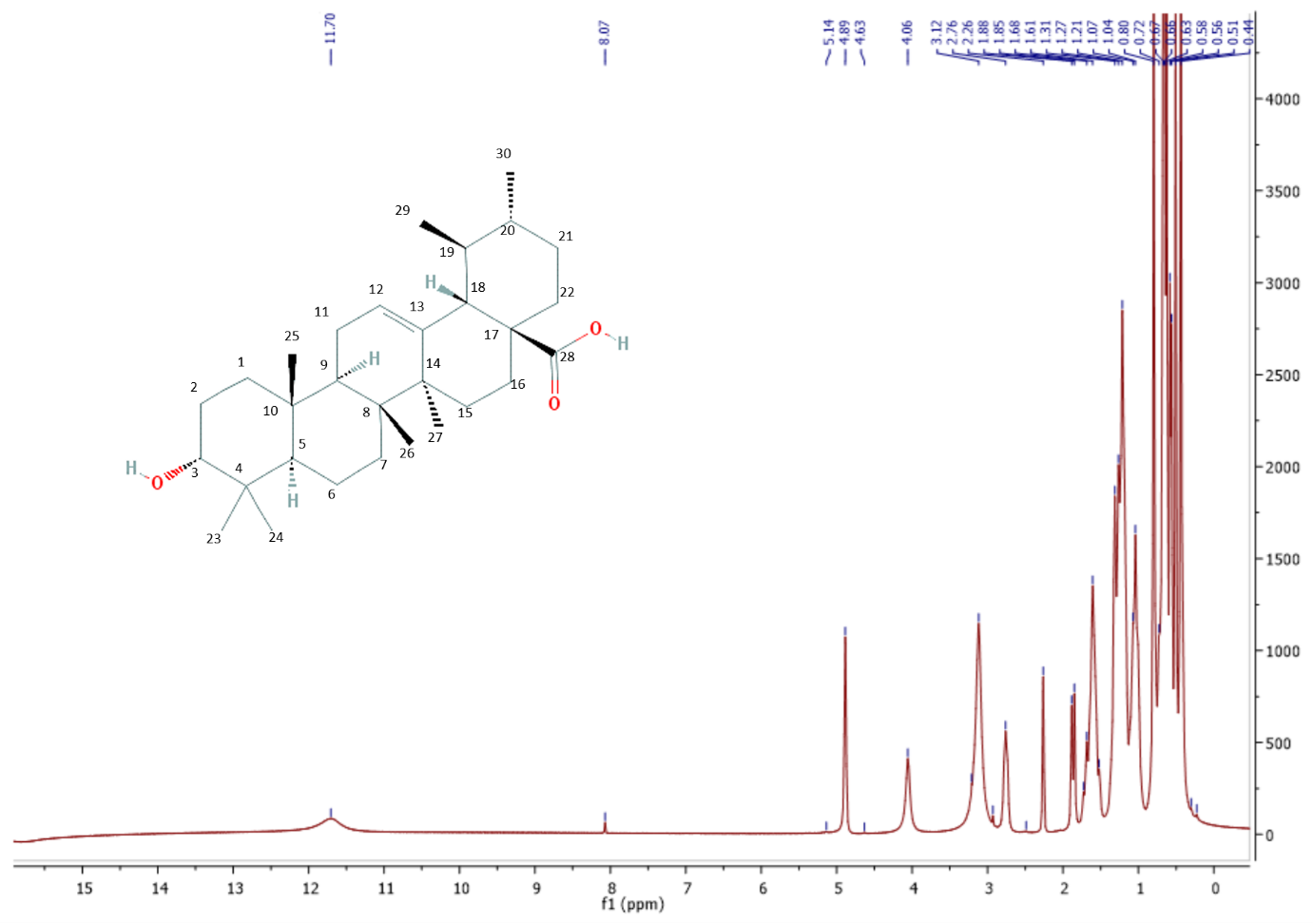


**Figure 7 - ^13^C NMR spectrum (δ, DMSO-d_6_, 75 MHz) of ursolic acid (UA)**


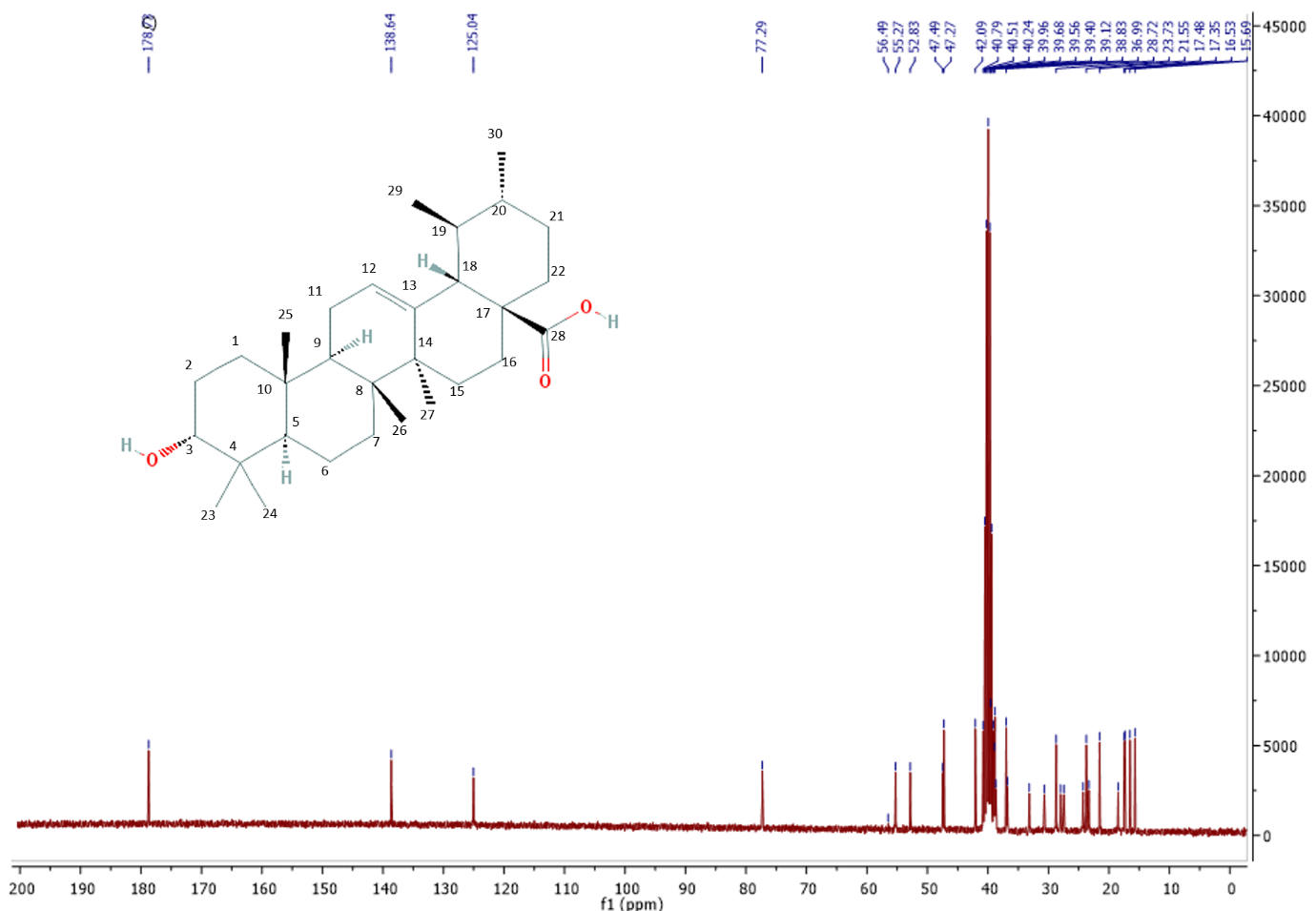


**Table 2 - Elemental analysis results**

| **Triterpene** | **Molecular formula** | **%C** | | **%H** | |
| --- | --- | --- | --- | --- | --- |
|  |  | *Theorical* | *Experimental* | *Theorical* | *Experimental* |
| Betulin | C_30_H_50_O_2_ | 81.4 | 81.2 | 11.4 | 11.2 |
| Lupeol | C_30_H_50_O | 84.4 | 84.3 | 11.8 | 11.6 |
| Ursolic acid | C_30_H_48_O_3_ | 78.9 | 79.0 | 10.6 | 10.5 |
